# Development and validation of a diagnostic and prognostic model for lung metastasis of hepatocellular carcinoma: a study based on the SEER database

**DOI:** 10.3389/fmed.2023.1171023

**Published:** 2023-07-19

**Authors:** Guangzhao Shao, Yao Zhi, Zhongqi Fan, Wei Qiu, Guoyue Lv

**Affiliations:** Department of Hepatobiliary and Pancreatic Surgery, First Hospital of Jilin University, Changchun, Jilin, China

**Keywords:** hepatocellular carcinoma, lung metastasis, nomogram, prognosis, SEER

## Abstract

**Background:**

Lung metastasis (LM) is a common occurrence in patients with hepatocellular carcinoma (HCC), and it is associated with a poorer prognosis compared to HCC patients without LM. This study aimed to identify predictors and prognostic factors for LM in HCC patients as well as develop diagnostic and prognostic nomograms specifically tailored for LM in HCC patients.

**Methods:**

A retrospective analysis was conducted on HCC patients from the Surveillance, Epidemiology, and End Results (SEER) database, covering the period from 2010 to 2015. The study employed multivariate logistic regression analysis to identify risk factors associated with LM in HCC patients. Additionally, multivariate Cox proportional hazards regression analysis was utilized to investigate prognostic factors for HCC patients with LM. Subsequently, two nomograms were developed to predict the risk and prognosis of LM in HCC patients. The performance of the nomograms was evaluated through calibration curves, receiver operating characteristic (ROC) curves, and decision curve analysis (DCA).

**Result:**

This retrospective study included a total of 5,934 patients diagnosed with HCC, out of which 174 patients were diagnosed with LM. Through multivariate logistic regression analysis, several independent risk factors for LM in HCC patients were identified, including tumor grade, tumor size, American Joint Committee for Cancer (AJCC) T stage, and AJCC N stage. Furthermore, multivariate Cox analysis revealed that tumor grade, delayed treatment, surgery, and radiation were independent prognostic factors for HCC patients with LM. To assess the predictive power of the developed nomograms, calibration curves, receiver operating characteristic (ROC) analysis, and decision curve analysis (DCA) were employed. The findings demonstrated that the nomograms exhibited satisfactory performance in both the training and validation sets. Additionally, the prognostic nomogram effectively stratified HCC patients with LM into low- and high-risk groups for mortality.

**Conclusion:**

These two nomograms optimally predicted the risk and prognosis of LM in HCC patients. Both nomograms have satisfactory performance. This would help clinicians to make accurate clinical decisions.

## 1. Introduction

Hepatocellular carcinoma (HCC) represents a prevalent malignancy globally, with a significant impact on public health. In 2020, an estimated 9,05,677 new cases of HCC were diagnosed worldwide, accompanied by approximately 8,30,180 reported deaths. The incidence of HCC is notably higher in transitioning countries, reflecting the dynamic nature of the disease burden across different regions. These statistics underscore the urgent need for effective strategies in the prevention, early detection, and treatment of HCC to mitigate its global impact (1). The mortality rate of HCC patients has decreased due to advances in early diagnosis and treatment. However, distant metastases still occur in a significant proportion (14.0%−36.7%) of HCC patients at the time of initial diagnosis (2, 3). The lungs are the predominant site of extrahepatic metastases in HCC patients, with a median survival of 8.1 months after the diagnosis of extrahepatic metastases. (4–6). Hence, it is imperative to identify risk factors associated with LM in HCC and develop diagnostic and prognostic models to effectively monitor high-risk subgroups. The traditional American Joint Committee for Cancer (AJCC) TNM staging system is still the most frequent tool for assessing the prognosis of cancer patients. It consists of three main components: tumor size, lymph node metastasis, and distant metastasis, but the TNM staging system cannot accurately measure the risk for patients with distant metastatic malignancies (7–9). A nomogram is a graphical depiction of a predictive model derived from individual predictive information that can be used to assess numerical probabilities of events, such as morbidity and mortality (10, 11). Nomograms have emerged as a valuable tool for clinicians in predicting the prognosis of malignant tumors, offering several advantages over traditional methods. In previous studies, numerous diagnostic and prognostic factors have been identified, further enhancing the applicability and reliability of nomograms in clinical practice (12, 13). Nevertheless, it should be noted that the variables included in these studies may not be ideal parameters for predicting LM in HCC. As a result, the clinical efficacy of existing models is somewhat limited. Furthermore, there is a scarcity of studies that specifically investigate the cancer-specific survival (CSS) of HCC patients, further underscoring the need for comprehensive research in this area.

Therefore, there is a necessity to fully comprehend the epidemiological characteristics of HCC patients with LM to identify risk and prognostic factors for LM. This study aimed to select demographic and clinicopathological data from the Surveillance, Epidemiology, and End Results (SEER) database to develop diagnostic and prognostic nomograms to determine the risk and prognosis of LM in HCC patients.

## 2. Patients and methods

### 2.1. Patients and variables inclusion

In this study, we included HCC patients from 2010 to 2015 in the SEER database. These data contained baseline demographics, tumor characteristics, treatment options, and survival time. The inclusion criteria were as follows: (1) International Classification of Diseases for Oncology, third edition [ICD-O-3] code 8170 to 8175 and (2) diagnosed between 2010 and 2015. The exclusion criteria were as follows: (1) the presence of primary tumors at other sites; (2) patients lacking important clinicopathological information; and (3) survival time of <1 month. Finally, 5,934 HCC patients were enrolled in this study. The following variables were analyzed to determine the risk factors of LM from HCC: age, sex, race, grade, tumor size, AJCC T stage, AJCC N stage, and alpha-fetoprotein (AFP). A total of 12 variables were analyzed to determine the prognosis for HCC patients with LM, including age, sex, race, income, marital status, grade, AJCC T stage, AJCC N stage, delayed treatment, surgery (performed or not performed), radiation (performed or not performed), and chemotherapy (performed or not performed). As our study used established data and did not involve interactions with human patients, institutional review board approval was not required. In addition, we used the seventh edition of the AJCC TNM staging system, which is available between 2010 and 2015.

### 2.2. Statistical analysis

All statistical analyses in our study were performed in SPSS 25.0 and R software (version 4.2.1). Using Python, all patients were randomly divided into training and validation sets in the ratio of 7:3. The chi-square test was applied to compare these variables between the training and validation sets. Significant variables (*P* < 0.05) from the univariate logistic analysis were included in multivariate binary logistic regression analysis to identify independent risk factors of LM in HCC patients. For prognostic factors, the univariate Cox regression analysis was used to identify prognostic variables. Significant variables (*P* < 0.1) were then included in the multivariate Cox regression analysis to determine the independent prognostic factors for HCC with LM. Diagnostic and prognostic nomograms were created based on the results of multivariate analysis. The receiver operating characteristic (ROC) curve for the diagnostic nomogram and the prognostic nomogram was created. The area under the curve (AUC) was used to assess the discrimination of these nomograms. By analyzing the ROC curves, the discriminative power of the diagnostic nomograms was also compared with the discriminative power of other independent risk factors. In addition, calibration curves and decision curve analysis (DCA) curves were created to evaluate these nomograms. Finally, all patients were divided into high- and low-risk groups according to the median risk score. The predictive value of the prognostic nomogram was verified using survival curves with the log-rank test. In this study, the primary outcome for prognostic survival was CSS, which was defined as the date from diagnosis to death (due to cancer cause) or to the last follow-up visit.

## 3. Result

### 3.1. Characteristics of HCC patients

**Figure 1:**
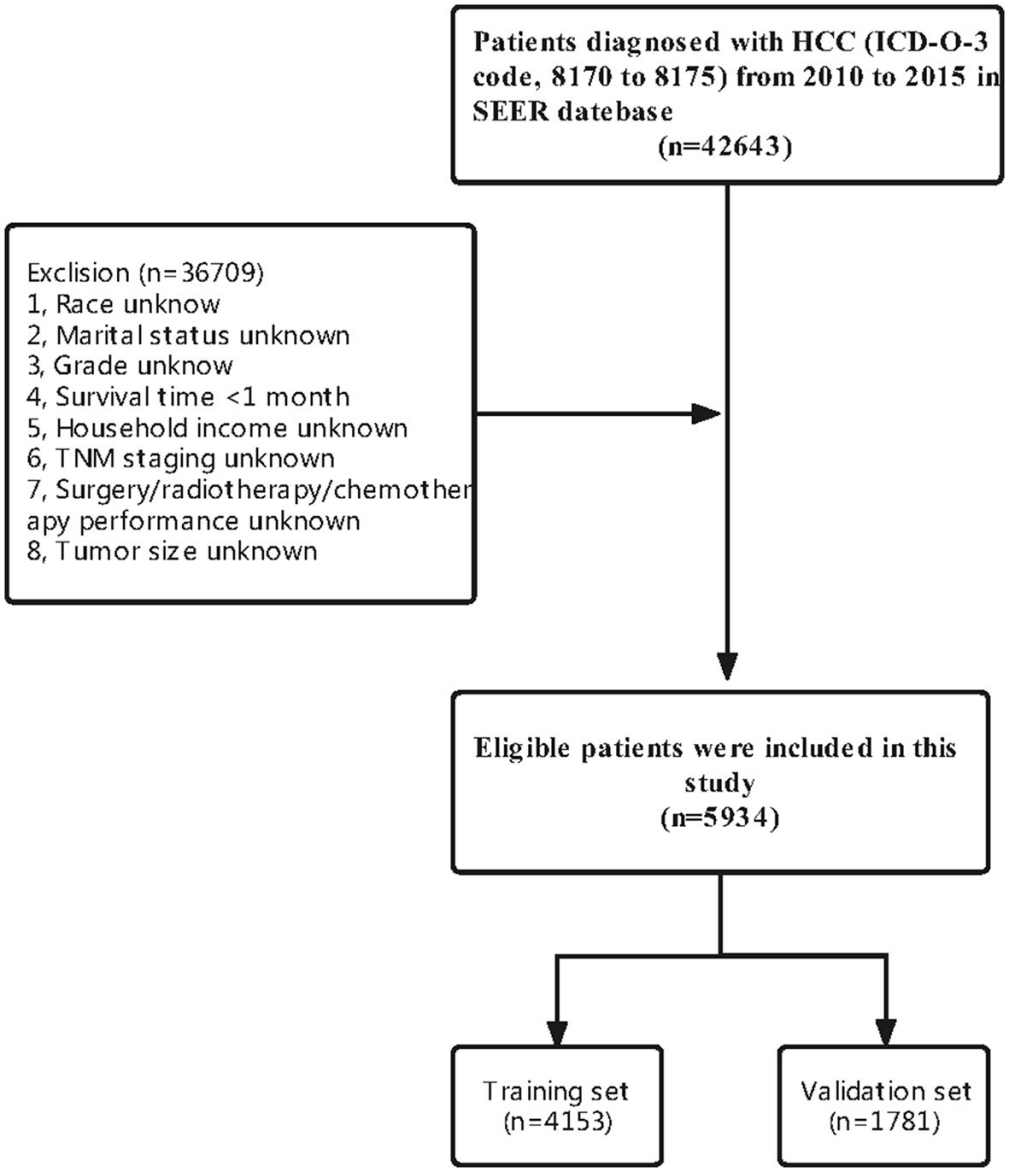
Flowchart of including and dividing patients.

A total of 5,934 HCC patients were included in this study according to our criteria. Meanwhile, 4153 (70%) patients were assigned to the training set and 1,781 (30%) patients were included in the validation set (Figure 1). There were no significant differences in most of the characteristics of patients between the training and validation sets (Table 1).

**Table 1:** Clinical and pathological features of HCC patients.

**Variables**	**Subgroup**	**Overall**	**Training set**	**Validation set**	***P*-value**
		**5,934**	**4,153**	**1,781**	
**Age, year (%)**					0.484
	≤ 50	424 (7.1)	286 (6.9)	138 (7.7)	
	50–70 (70)	3,871 (65.2)	2,713 (65.3)	1,158 (65.0)	
	>70	1,639 (27.6)	1,154 (27.8)	485 (27.2)	
**Sex (%)**					0.493
	Male	4,557 (76.8)	3,200 (77.1)	1,357 (76.2)	
	Female	1,377 (23.2)	953 (22.9)	424 (23.8)	
**Race (%)**					0.378
	White	3,949 (66.5)	2,763 (66.5)	1,186 (66.6)	
	Black	750 (12.6)	539 (13.0)	211 (11.8)	
	Other	1,235 (20.8)	851 (20.5)	384 (21.6)	
**Grade (%)**					0.277
	I	1,719 (29.0)	1,231 (29.6)	488 (27.4)	
	II	2,838 (47.8)	1,980 (47.7)	858 (48.2)	
	III	1,283 (21.6)	878 (21.1)	405 (22.7)	
	IV	94 (1.6)	64 (1.5)	30 (1.7)	
**Tumor Size, cm (%)**					0.048
	≤ 3	1,484 (25.0)	1,003 (24.2)	481 (27.0)	
	3–5 (5)	1,457 (24.6)	1,043 (25.1)	414 (23.2)	
	>5	2,993 (50.4)	2,107 (50.7)	886 (49.7)	
**AJCC T stage (%)**					0.813
	T1	2,745 (46.3)	1,917 (46.2)	828 (46.5)	
	T2	1,438 (24.2)	1,004 (24.2)	434 (24.4)	
	T3	1,505 (25.4)	1,053 (25.4)	452 (25.4)	
	T4	246 (4.1)	179 (4.3)	67 (3.8)	
**AJCC N stage (%)**					0.736
	N0	5,516 (93.0)	3,864 (93.0)	1,652 (92.8)	
	N1	418 (7.0)	289 (7.0)	129 (7.2)	
**Lung metastasis (%)**					0.822
	No	5,704 (96.1)	3,990 (96.1)	1,714 (96.2)	
	Yes	230 (3.9)	163 (3.9)	67 (3.8)	
**AFP (%)**					0.473
	Negative	1,918 (32.3)	1,330 (32.0)	588 (33.0)	
	Positive	4,016 (67.7)	2,823 (68.0)	1,193 (67.0)	

HCC, hepatocellular carcinoma; Other, Asian/Pacific Islander, American Indian/Alaskan Native; AJCC, American Joint Committee for Cancer; AFP, alpha-fetoprotein.

### 3.2. Risk factors of LM in HCC patients

To determine these variables associated with LM in HCC patients, these variables with a *P*-value of < 0.05 in the univariate analysis were included in the multivariate logistic regression analysis. The result showed that grade (*P* < 0.001), tumor size (*P* < 0.001), AJCC T stage (*P* = 0.003), and AJCC N stage (*P* < 0.001) were independent predictors of LM in HCC patients (Table 2).

**Table 2:** Logistic analysis of risk factors of LM in HCC patients.

**Variables**	**Subgroup**	**Univariate**		**Multivariate**	
		**OR 95 % CI**	***P*-value**	**OR 95 % CI**	***P*-value**
**Age, year**					
	≤ 50	Reference	0.673		
	50–70 (70)	1.239 (0.620, 2.475)	0.544		
	>70	1.365 (0.662, 2.812)	0.399		
**Sex**					
	Male	Reference	0.403		
	Female	0.847 (0.574, 1.250)			
**Race**					
	White	Reference	0.908		
	Black	1.106 (0.698, 1.753)	0.667		
	Other	1.001 (0.673, 1.491)	0.995		
**Grade**					
	I	Reference	< 0.001	Reference	< 0.001
	II	1.297 (0.821, 2.047)	0.265	1.129 (0.708, 1.8)	0.61
	III	3.838 (2.458, 5.992)	< 0.001	2.288 (1.428, 3.666)	0.001
	IV	3.641 (1.357, 9.768)	0.010	2.054 (0.747, 5.649)	0.163
**Tumor size, cm**					
	≤ 3	Reference	< 0.001	Reference	< 0.001
	3–5 (5)	1.138 (0.508, 2.553)	0.753	1.036 (0.46, 2.334)	0.931
	>5	6.370 (3.431, 11.824)	0.000	3.779 (1.951, 7.323)	< 0.001
**AJCC T stage**					
	T1	Reference	< 0.001	Reference	0.003
	T2	0.821 (0.477, 1.414)	0.477	1.022 (0.582, 1.795)	0.939
	T3	3.264 (2.232, 4.774)	< 0.001	1.473 (0.973, 2.229)	0.067
	T4	6.910 (4.118, 11.596)	< 0.001	2.796 (1.602, 4.881)	< 0.001
**AJCC N stage**					
	N0	Reference	< 0.001	Reference	< 0.001
	N1	5.454 (3.761, 7.907)		2.876 (1.935, 4.275)	
**AFP**					
	Negative	Reference	0.002	Reference	0.231
	Positive	1.825 (1.244, 2.679)		1.281 (0.854, 1.92)	

LM, lung metastasis; HCC, hepatocellular carcinoma; OR, odds ratio; CI, confidence interval; Other, Asian/pacific islander, American Indian/Alaskan Native; AJCC, American Joint Committee for Cancer; AFP, alpha-fetoprotein.

### 3.3. Diagnostic nomogram development and validation

**Figure 2:**
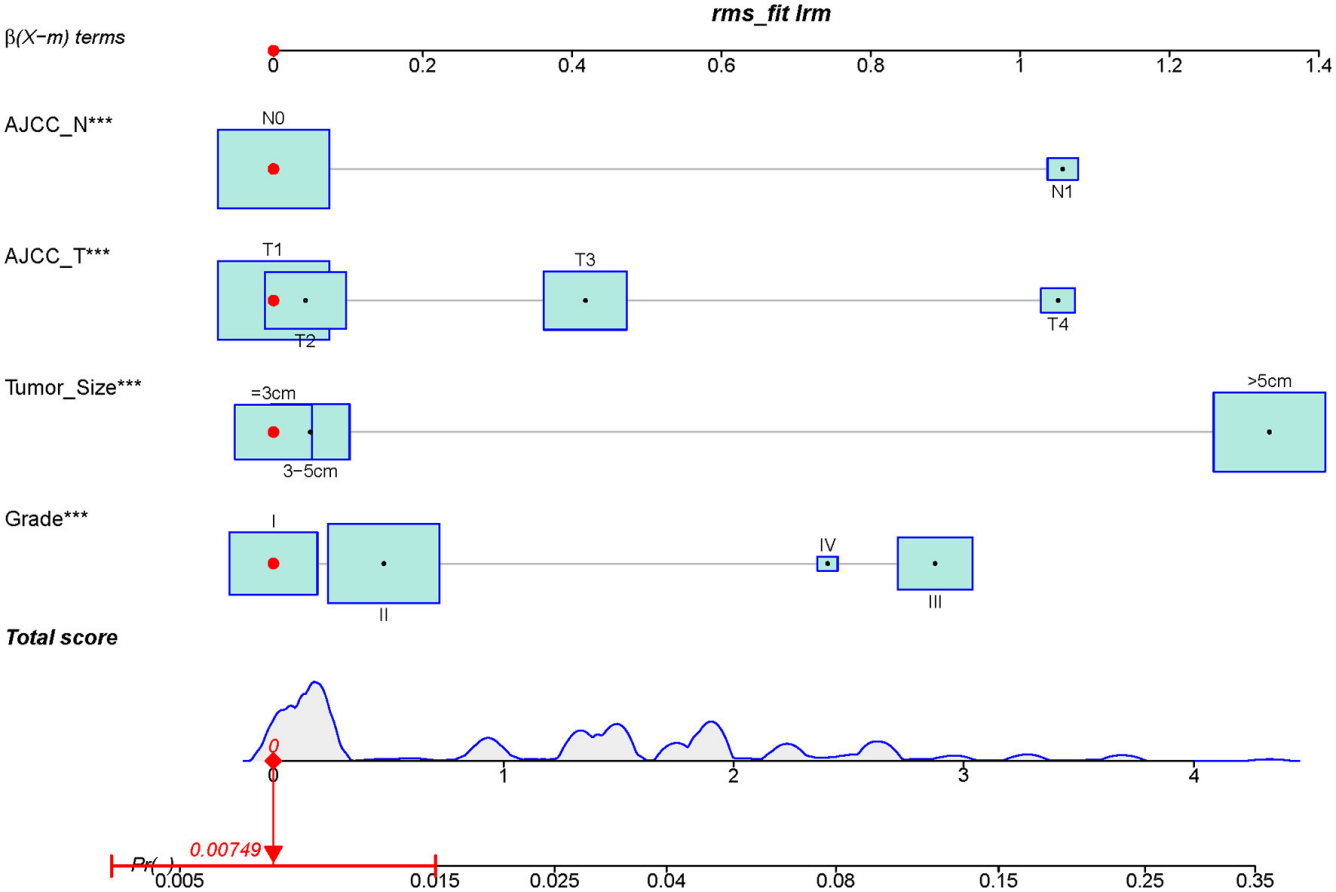
Diagnostic nomogram for predicting LM from HCC patients.

**Figure 3:**
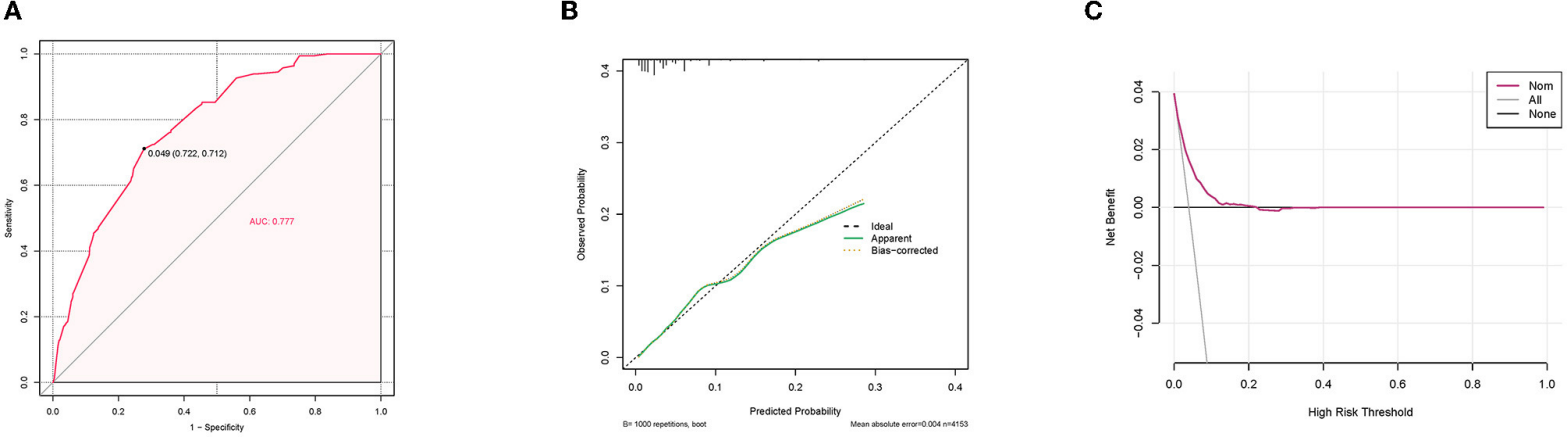
Receiver operating characteristic curve **(A)**, calibration curve **(B)**, and decision curve analysis **(C)** of the training set.

**Figure 4:**
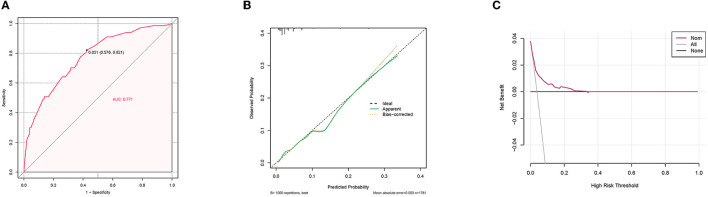
Receiver operating characteristic curve **(A)**, calibration curve **(B)**, and decision curve analysis **(C)** of the validation set.

**Figure 5:**
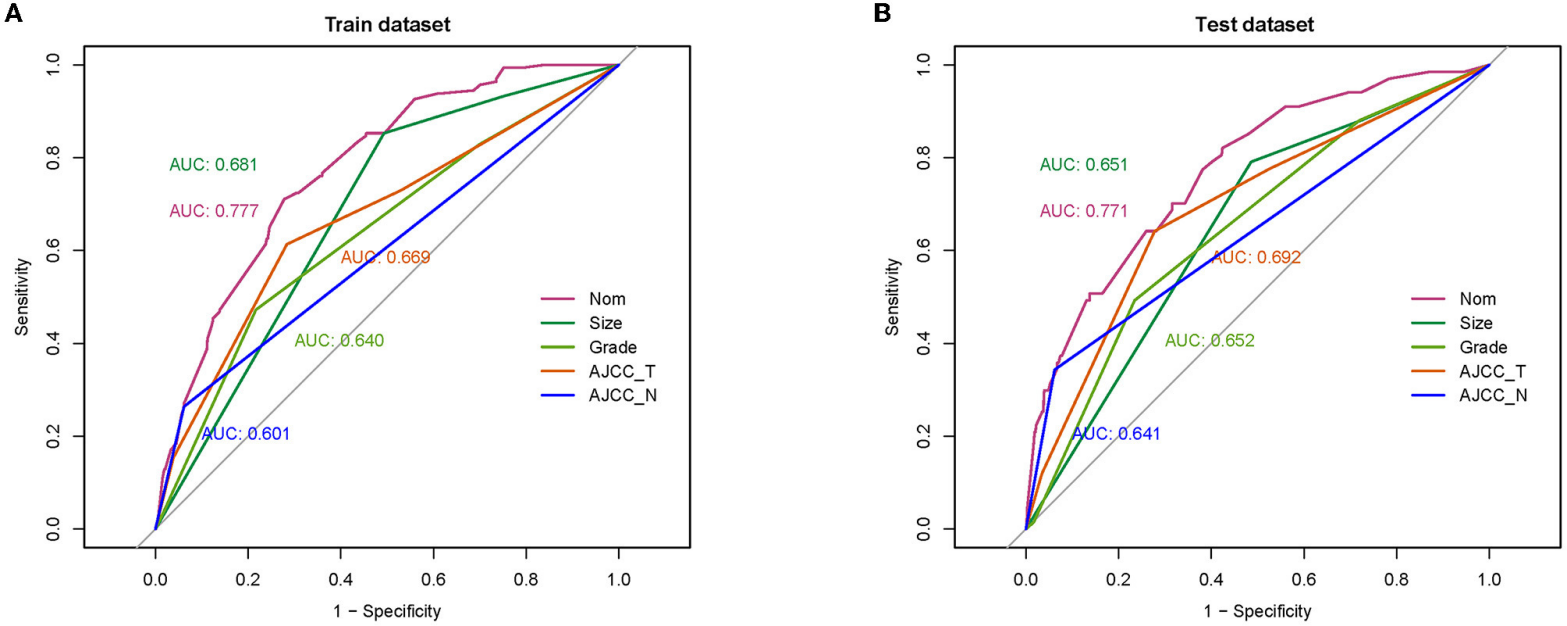
Comparison of area under the receiver operating characteristic curve between diagnostic nomogram and each independent predictor in the training set **(A)** and the validation set **(B)**.

A diagnostic nomogram was established for LM risk assessment in HCC patients based on independent predictors (Figure 2). ROC analysis showed that the AUCs of the diagnostic nomogram reached 0.777 in the training set and 0.771 in the validation set (Figures 3A, 4A). Meanwhile, in both the training and validation sets, the calibration curves showed that the actual observations were in high agreement with the predicted results of the diagnostic nomogram, and the DCA indicated that the diagnostic nomogram could be a good diagnostic tool for LM in HCC patients in clinical practice (Figures 3B, C, 4B, C). Furthermore, the result showed that the AUCs of all independent predictors were lower than the AUCs of the diagnostic nomograms in both the training and validation sets (Figure 5).

### 3.4. Characteristics of HCC patients with LM

A total of 174 eligible patients were used to study prognostic factors. All patients were randomly categorized into a training set (*n* = 121) and a validation set (*n* = 53). Most of the variables were not found to be significantly different between the training and validation sets (Table 3).

**Table 3:** Clinical and pathological features of HCC patients with LM.

**Variables**	**Subgroup**	**Overall**	**Training set**	**Validation set**	***P*-value**
		**174**	**121**	**53**	
**Age, year (%)**					0.481
	≤ 60	80 (46.0)	53 (43.8)	27 (50.9)	
	>60	94 (54.0)	68 (56.2)	26 (49.1)	
**Sex (%)**					1.000
	Male	139 (79.9)	97 (80.2)	42 (79.2)	
	Female	35 (20.1)	24 (19.8)	11 (20.8)	
**Race (%)**					0.105
	White	111 (63.8)	78 (64.5)	33 (62.3)	
	Black	23 (13.2)	12 (9.9)	11 (20.8)	
	Other^a^	40 (23.0)	31 (25.6)	9 (17.0)	
**Income (%)**					0.042
	≤ 55,000	52 (29.9)	30 (24.8)	22 (41.5)	
	>55,000	122 (70.1)	91 (75.2)	31 (58.5)	
**Marital status (%)**					0.070
	Married	99 (56.9)	74 (61.2)	25 (47.2)	
	Unmarried	35 (20.1)	25 (20.7)	10 (18.9)	
	Other^b^	40 (23.0)	22 (18.2)	18 (34.0)	
**Grade (%)**					0.364
	I / II	96 (55.2)	70 (57.9)	26 (49.1)	
	III / IV	78 (44.8)	51 (42.1)	27 (50.9)	
**AJCC T stage (%)**					0.741
	T1	47 (27.0)	34 (28.1)	13 (24.5)	
	T2	20 (11.5)	12 (9.9)	8 (15.1)	
	T3	80 (46.0)	57 (47.1)	23 (43.4)	
	T4	27 (15.5)	18 (14.9)	9 (17.0)	
**AJCC N stage (%)**					0.344
	N0	121 (69.5)	81 (66.9)	40 (75.5)	
	N1	53 (30.5)	40 (33.1)	13 (24.5)	
**Delayed treatment, month (%)**					0.374
	≤ 1	125 (71.8)	84 (69.4)	41 (77.4)	
	>1	49 (28.2)	37 (30.6)	12 (22.6)	
**Surgery (%)**					1.000
	No	155 (89.1)	108 (89.3)	47 (88.7)	
	Yes	19 (10.9)	13 (10.7)	6 (11.3)	
**Radiation (%)**					0.633
	No	139 (79.9)	95 (78.5)	44 (83.0)	
	Yes	35 (20.1)	26 (21.5)	9 (17.0)	
**Chemotherapy (%)**					0.768
	No	29 (16.7)	19 (15.7)	10 (18.9)	
	Yes	145 (83.3)	102 (84.3)	43 (81.1)	

HCC, hepatocellular carcinoma; LM, lung metastasis; Other^a^, Asian/Pacific Islander, American Indian/Alaskan Native; Other^b^, single, separated, divorced, widowed, and domestic partner; AJCC, American Joint Committee for Cancer.

### 3.5. Prognostic factors for HCC patients with LM

In the training set, these variables (grade, *P* = 0.002; AJCC T stage, *P* = 0.153; AJCC N stage, *P* = 0.012; delayed treatment, *P* = 0.022; radiation, *P* = 0.093) in the univariate Cox proportional hazards regression analysis were included in the multivariate Cox proportional hazards regression analysis. The result revealed that grade (*P* < 0.002), delayed treatment (*P* < 0.017), and radiation (*P* = 0.041) were identified as independent prognostic factors (Table 4).

**Table 4:** Univariate and multivariate Cox analysis in HCC patients with LM.

**Variables**	**Subgroup**	**Univariate**		**Multivariate**	***P*-value**
		**HR 95 % CI**	***P*** **-value**	**HR 95 % CI**	
**Age, year**					
	≤ 50	Reference	0.917		
	>50	1.019 (0.709, 1.467)			
**Sex**					
	Male	Reference	0.197		
	Female	1.345 (0.857, 2.111)			
**Race**					
	White	Reference	0.952		
	Black	1.100 (0.596, 2.029)	0.760		
	Other	0.996 (0.652, 1.523)	0.987		
**Income**					
	≤ 55,000	Reference	0.429		
	> 55,000	1.191 (0.773, 1.836)			
**Marital status**					
	Married	Reference	0.202		
	Unmarried	0.877 (0.556, 1.383)	0.571		
	Other	0.640 (0.392, 1.046)	0.075		
**Grade**					
	I/II	Reference	0.002	Reference	0.002
	III/IV	1.808 (1.247, 2.623)		1.813 (1.244, 2.642)	
**AJCC T stage**					
	T1	Reference	0.153		
	T2	1.668 (0.851, 3.270)	0.136		
	T3	1.358 (0.879, 2.097)	0.168		
	T4	1.916 (1.056, 3.477)	0.032		
**AJCC N stage**					
	N0	Reference	0.012		
	N1	1.661 (1.120, 2.465)			
	**Delayed treatment, month**					
	≤ 1	Reference	0.022	Reference	0.017
	>1	0.632 (0.427, 0.935)		0.614 (0.412, 0.915)	
**Surgery**					
	No	Reference	0.118		
	Yes	0.619 (0.340, 1.129)			
**Radiation**					
	No	Reference	0.093	Reference	0.041
	Yes	0.683 (0.438, 1.065)		0.691 (0.440, 1.087)	
**Chemotherapy**					
	No	Reference	0.883		
	Yes	1.039 (0.628, 1.717)			

HCC, hepatocellular carcinoma; LM, lung metastasis; Other ^a^Asian/Pacific Islander, American Indian/Alaskan Native; HR, hazard Ratio; CI, Confidence interval; Other ^b^single, separated, divorced, widowed, and domestic partner; AJCC, American Joint Committee for Cancer.

### 3.6. Prognostic nomogram development and validation

**Figure 6:**
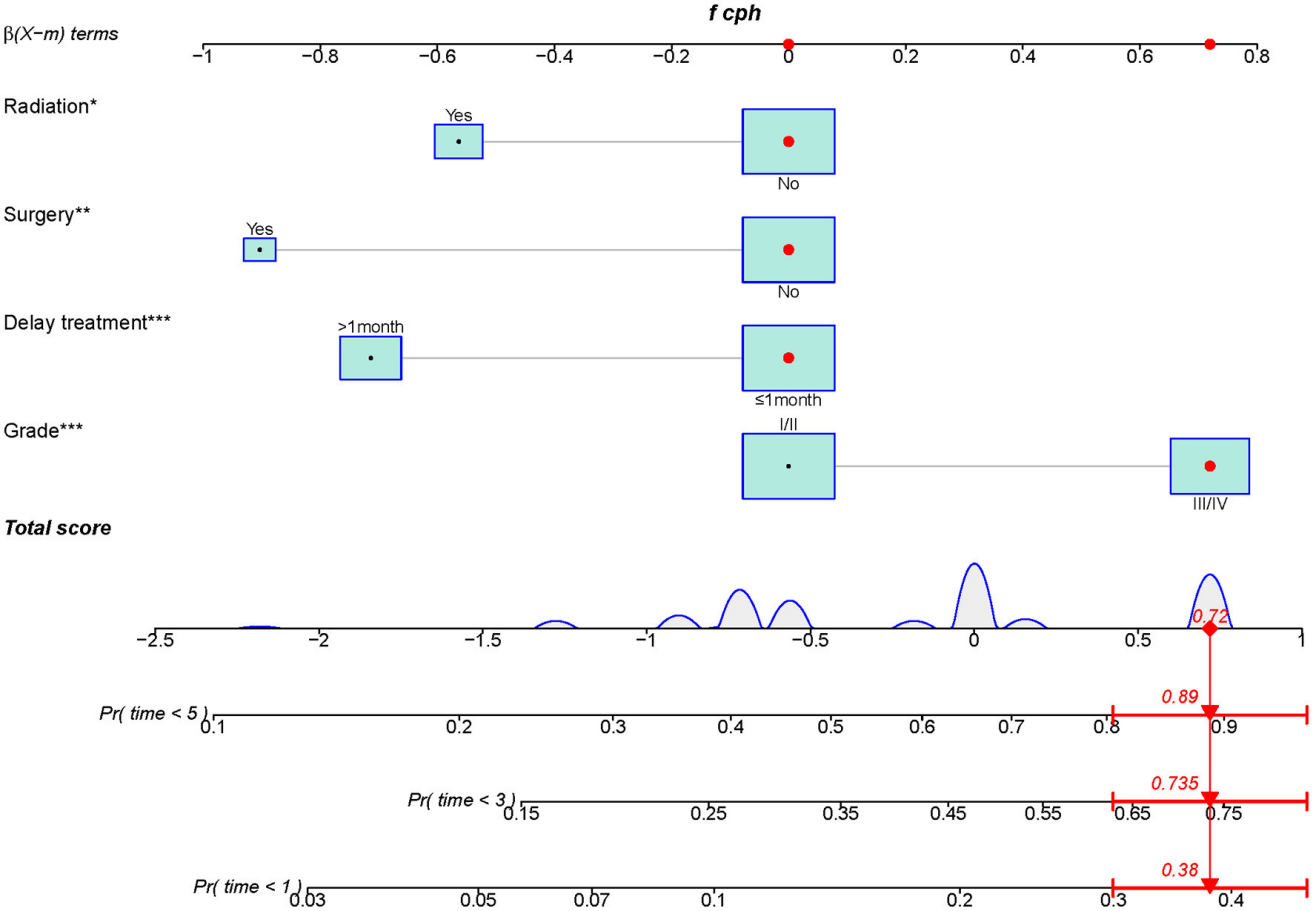
Prognostic nomogram for HCC patients with LM.

**Figure 7:**
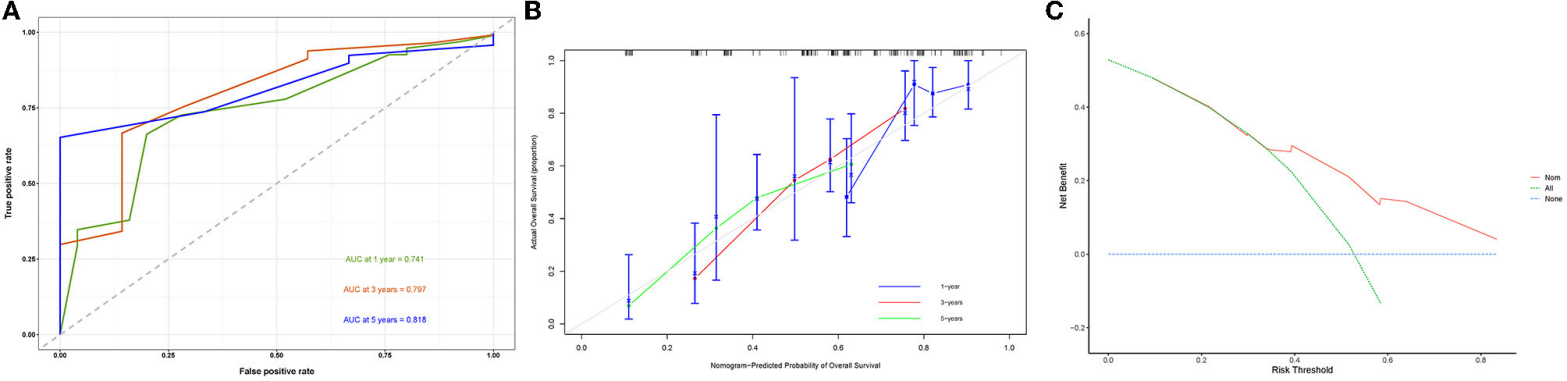
Receiver operating characteristic curve **(A)**, calibration curve **(B)**, and the decision curve analysis **(C)** of the training set.

**Figure 8:**
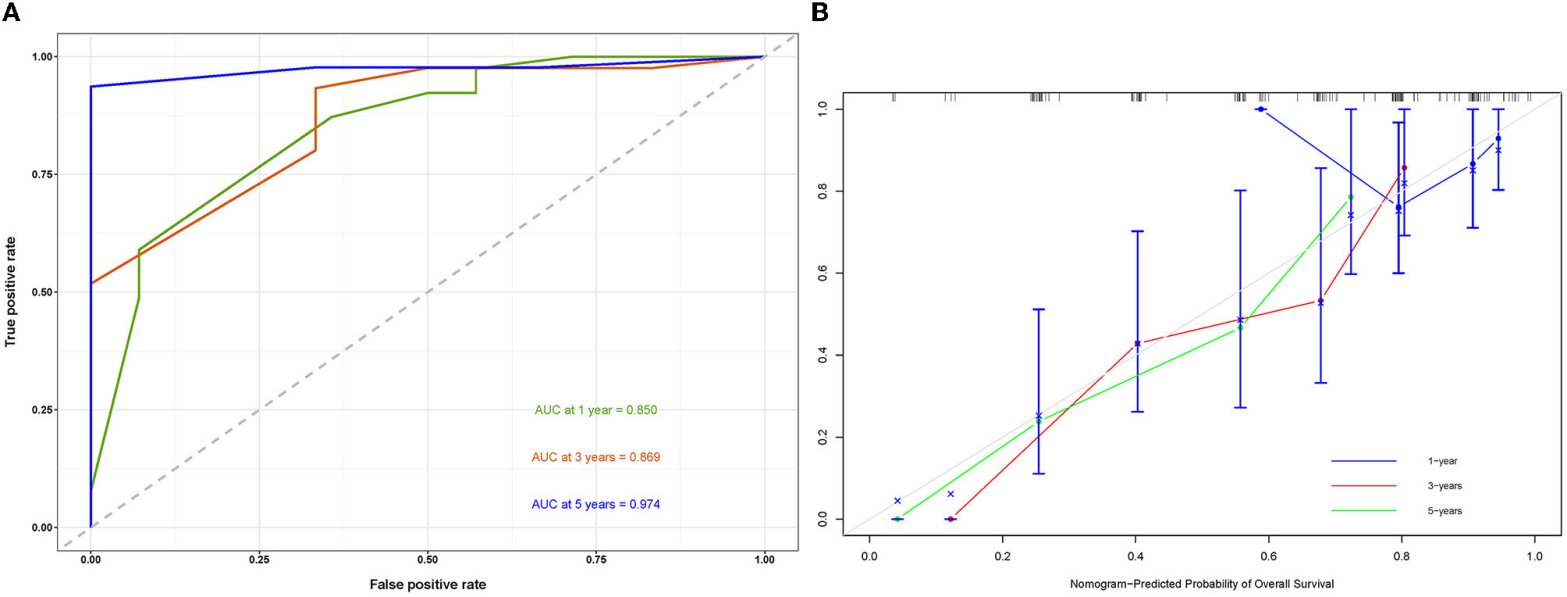
Receiver operating characteristic curve **(A)** and a calibration curve of the validation set **(B)**.

**Figure 9:**
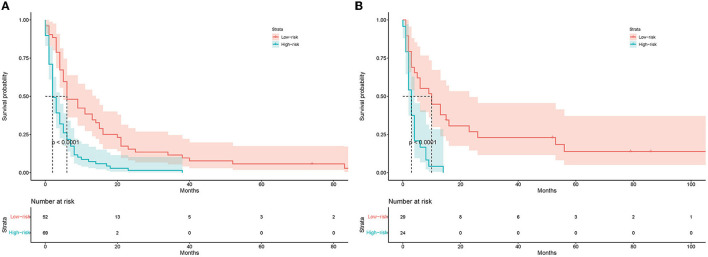
Kaplan–Meier survival curve of the training set **(A)** and the validation set **(B)**.

A prognostic nomogram was created based on the independent prognostic factors identified in the training set (Figure 6). The addition of surgery as an important treatment variable to the model can increase the interpretability of the predicted outcomes. The surgical coefficients in the model allow us to assess the degree of impact of different surgical procedures on the predicted outcomes and provide valuable information for clinical decision-making. Therefore, we chose to include surgery in the model to further improve predictive performance. The ROC analysis demonstrated promising performance of the prognostic nomogram in predicting 1-, 3-, and 5-year CSS. In the training set, the AUC values for the aforementioned time points were 0.741, 0.797, and 0.818, respectively (Figure 7A). The validation cohort further validated the robustness of the nomogram, with AUC values of 0.850, 0.869, and 0.974 for the corresponding time points (Figure 8A). The calibration curves also showed a good agreement between the prognostic nomogram predicting CSS and actual outcomes (Figures 7B, 8B). In addition, as shown by the DCA, the prognostic nomogram showed significant positive net benefits over a wide range of mortality risks, indicating that the prognostic nomogram had strong predictive efficiency and good clinical significance in predicting CSS for HCC patients with LM (Figure 7C). For data reasons, it is regrettably not possible to derive a decision curve of the validation set. In addition, we classified all patients into high-risk and low-risk groups based on the median of risk score (Figures 9A, B), and Kaplan–Meier survival curves showed that patients in the high-risk group had a worse prognosis than those in the low-risk group (Figures 9A, B).

## 4. Discussion

HCC is an aggressive tumor and the leading cause of cancer deaths worldwide. Approximately 8,00,000 people dead from HCC in 2020 (1). With the continuous advancement of early diagnosis and comprehensive treatment, the mortality rate of HCC patients has decreased compared with that before (14). Distant metastasis becomes a major cause of death in HCC patients (15). A study reported that 14%−36.7% of HCC patients will develop extrahepatic metastasis, and the lungs are the most common site of metastasis (16). Therefore, it is necessary to clarify the risk and prognostic factors of HCC patients with LM and to develop simple and reliable tools to facilitate early diagnosis of LM and to assist clinicians in making rational clinical decisions.

Although the predictive and prognostic factors of HCC with LM have been reported previously, the lack of important clinical and biochemical indicators, such as AFP, and extrahepatic metastasis such as bone metastasis and brain metastasis are not ideal parameters to predict LM of HCC (17). In this study, we selected variables that were more closely related to clinical practice, and independent risk factors and independent prognostic factors were derived by multivariate binary logistic regression analysis and the multivariate Cox regression analysis. We also created two nomograms based on these independent risk factors to predict the probability of LM in HCC patients and the prognosis of HCC patients with LM. Nomograms in our study with higher prognostic values compared to previous studies (17, 18). Both in the training and validation sets, the two nomograms showed high consistency between the predicted results and those observed in the clinic, which allows clinicians to make more accurate clinical decisions based on several easily available clinical data.

There is no doubt that early detection of LM is extremely important to prolong the survival time of HCC patients. At present, there are many studies on LM in HCC patients. Some studies reported that circASAP1 and miR-1247–3p promoted tumor cell growth and LM and were risk factors for LM in HCC patients (19, 20). However, these indicators are not clinically generalizable. Other studies reported that tumor size was an independent risk factor for LM in HCC patients (4, 21, 22). This was consistent with the results of our study. In general, large tumors grow in the body for a longer time and are prone to vascular invasion and metastasis. Ischemic necrosis is likely to occur within large tumors. A significant correlation was reported between the incidence of primary tumor metastasis and the degree of ischemic necrosis (22–24). Meanwhile, transcatheter chemoembolization is a common treatment for large tumors (25, 26). There was evidence suggesting that transcatheter chemoembolization may lead to tumor dissemination, with the lungs being one of the most common sites of dissemination (27, 28). Previous studies reported that AFP was positively associated with the risk of LM in HCC patients (29, 30). However, in our study, AFP was not an independent risk factor for LM in HCC patients. We believed that an AFP value >400 was a more desirable cutoff value for predicting LM in HCC patients (31). Unfortunately, we cannot get such data from the SEER database. In our study, grade, tumor size, AJCC T stage, and AJCC N stage were significant predictors of LM in HCC patients, and previous studies also confirmed that these factors were correlated with extrahepatic metastases (32, 33). Meanwhile, the result showed that the discriminatory power of the predicted nomogram was stronger than any other individual predictor. This indicated the advantage of the synthetic prediction model. Currently, CT scanning is commonly used to detect lung metastases, but this imaging technique is inadequate for early metastatic lesions in the lung, and computer-aided detection of lung nodules (CAD) has shown great advantages in diagnosing lung nodules, especially small and isolated nodules. We therefore recommend regular screening for lung nodules and, if necessary, lung biopsy in HCC patients with high-risk LM factors.

We also found that grade, delayed treatment, and radiation were independent prognostic factors for HCC patients with LM in this study. Based on the above prognostic factors, we developed the prognostic nomogram. Due to the importance of surgery in clinical practice, we chose to include it in the model to further improve predictive performance. The prognostic nomogram performed effectively in both the training and validation sets and could be used as an intuitive and effective tool for identifying patients with high-risk factors. Previous studies indicated that HCC patients with extrahepatic metastases had an extremely poor prognosis, with a median survival time of 5.9 months (34). Although the prognosis of HCC patients with LM remains poor, early detection and timely appropriate treatment are essential to improve the prognosis of patients. In our study, radiations were independent protective factors for CSS in HCC patients with LM. This was consistent with the results of some previous studies (35, 36). Chemotherapy was one of the recommended treatments for HCC patients with extrahepatic metastases, and previous studies reported that sorafenib, adriamycin, and gemcitabine had a positive impact on survival in advanced hepatoma (37–40). Contrary to our expectation, chemotherapy was not an independent prognostic factor for HCC patients with LM in this study. Unfortunately, we were unable to conduct a detailed study of the prognostic impact of each specific chemotherapy regimen because we did not have access to specific information about chemotherapy regimens. Therefore, we suggest that for a good prognosis, clinical treatment in HCC patients with LM could tend to be surgery and radiation therapy. Notably, previous studies showed that once a tumor had distant organ metastasis, it may accelerate the metastasis to other organs, and the number of metastatic organs also had a significant impact on survival (41). Therefore, we suggest that for HCC patients with LM, a detailed examination of other organs, such as the brain and bone, is necessary.

However, several limitations to our study should be noted. First, this was a retrospective study, and selection bias was inevitable. Second, the limited number of patients (*n* = 174) may lead to bias, and we should interpret the study results with caution. Third, we did not have access to specific information for patient treatment, such as specific chemotherapy regimens and surgical methods. Finally, due to the rarity of lung metastases from hepatocellular carcinoma, we were unable to perform further validation of the model using data from our own center. In future, we will focus on the prospective validation of the model and the inclusion of additional centers to verify the performance and stability of the model.

## 5. Conclusion

The two nomograms developed in this study can visually and effectively predict the risk of LM in HCC patients and assess the prognosis of HCC patients with LM. The validation set demonstrated the promising performance and clinical utility of the predictive model. This information can help clinicians to make accurate clinical decisions.

## Data availability statement

The original contributions presented in the study are included in the article/supplementary material, further inquiries can be directed to the corresponding author.

## Author contributions

GS conceived and designed the study, wrote the manuscript, and performed a literature search. GS and YZ generated the figures and tables. GS, ZF, and WQ analyzed the data. GL critically reviewed the manuscript and supervised the research. All authors have read and approved the manuscript.
